# Factors Influencing the Course of Hospitalization in Children with Respiratory Syncytial Virus Infection: A Retrospective Single-Center Study at the Department of Pediatrics, Wadowice Hospital, Poland

**DOI:** 10.3390/medicina62030455

**Published:** 2026-02-28

**Authors:** Klaudia Kasperek, Dominik Gałuszka, Agnieszka Sumara, Anna Kurkiewicz-Piotrowska

**Affiliations:** 1 Department of Pediatrics, Hospital in Wadowice, Karmelicka 5, 34-100 Wadowice, Poland; 2 Department of Cosmetology, Faculty of Medicine and Health Sciences, University of Applied Sciences in Tarnow, Mickiewicza 8, 33-100 Tarnów, Poland; 3 Department of Organization and Supervision Emergency Medicine Department in Tarnow, Fatimskiej 2, 33-100 Tarnów, Poland; 4 Department of Chemistry and Biochemistry, Institute for Basic Sciences, Faculty of Physiotherapy, University of Physical Culture in Krakow, Jana Pawła II 78, 31-571 Kraków, Poland

**Keywords:** respiratory syncytial virus infections, hospitalization, pediatrics, risk factors, disease severity

## Abstract

*Background and Objectives*: Respiratory syncytial virus (RSV) is a leading cause of hospitalization among infants and young children. The clinical course of RSV infection varies considerably depending on age and selected clinical factors. The objective of this study was to identify demographic and clinical variables associated with the course of hospitalization in children admitted due to laboratory-confirmed RSV infection. *Materials and Methods*: A retrospective observational study was conducted based on the medical records of 100 immunocompetent pediatric patients hospitalized due to RSV infection in the Department of Pediatrics of Hospital in Wadowice, Poland, between December 2021 and April 2023. Inclusion criteria were age ≤ 5 years and laboratory-confirmed RSV infection. Patients with congenital heart disease, chronic lung disease (including cystic fibrosis), immunodeficiency, or other severe chronic conditions were excluded. Collected data included age, gestational age at birth, mode of delivery, vaccination status, clinical presentation, length of hospital stay, C-reactive protein (CRP) levels, seasonality of infection, and use of antibiotic therapy. *Results*: The median length of hospitalization was 6 days (range: 0–18). Younger age was significantly associated with longer hospital stay (*p* < 0.05) and higher CRP levels (*p* < 0.05). No significant associations were observed between hospitalization duration and mode of delivery or vaccination status. Gestational age at birth did not influence the number of clinical symptoms. The need for antibiotic therapy differed significantly according to the season of infection (*p* < 0.05). *Conclusions*: In children hospitalized with RSV infection, age and seasonality were the primary factors influencing the course of hospitalization, whereas perinatal factors such as mode of delivery and vaccination status had no significant impact. These findings underscore the importance of age-oriented clinical assessment and support efforts to optimize antimicrobial stewardship during RSV seasons.

## 1. Introduction

Respiratory syncytial virus (RSV) is the most frequently identified etiological agent of respiratory tract infections in the pediatric population, particularly among neonates and children under 2 years of age [1]. RSV accounts for a substantial proportion of hospitalizations of infants and young children due to respiratory infections, especially bronchiolitis and pneumonia. In addition, older adults and immunocompromised individuals are also at increased risk of severe complications associated with RSV infection [2]. RSV infections are characterized by a marked seasonality of transmission. The peak incidence occurs during the winter and early spring months, which are associated with higher humidity and lower temperatures that favor viral survival in the environment, as well as increased aggregation of children in enclosed spaces [3]. RSV is highly contagious and spreads primarily via respiratory droplets and direct contact with respiratory secretions, facilitating transmission in childcare settings such as nurseries and kindergartens, as well as within households [4].

Initial symptoms of RSV infection do not differ significantly from those of typical viral upper respiratory tract infections; however, in the youngest children, the inflammatory process frequently progresses to bronchiolitis and pneumonia, which constitute the main causes of respiratory failure and indications for hospitalization. The most commonly observed symptoms include rhinorrhea, cough, fever, fatigue, dyspnea, breathing difficulties, and feeding problems, resulting from airway edema and obstruction that limit airflow to the lungs [1].

Age is a key determinant of the clinical severity of RSV infection. The highest risk of severe disease is observed in infants younger than 6 months, in whom rapid respiratory decompensation may occur [5]. This is attributable to limited ventilatory reserve, small airway caliber, immaturity of the immune system, and an inability to effectively clear respiratory secretions. Consequently, bronchiolar obstruction caused by mucus plugging, mucosal edema, and impaired ciliary function rapidly leads to hypoxia. Progressive dyspnea then becomes the predominant symptom and often determines the need for hospitalization in the youngest patients [6].

Preterm infants constitute a particularly vulnerable group due to impaired lung development and delayed immune maturation. They have narrower airways and a reduced respiratory reserve; therefore, edema and excessive mucus production during RSV infection significantly compromise ventilation [7]. Increased susceptibility to infection results in more severe and prolonged disease episodes, frequently requiring oxygen therapy and, in some cases, respiratory support. An additional risk factor in this group is frequent hospitalization during the perinatal period, which increases exposure to RSV in the hospital environment [8].

Comorbidities also have a significant impact on the course of hospitalization, particularly conditions affecting the respiratory system, such as asthma and cystic fibrosis, as well as cardiovascular diseases, including congenital heart defects. Children with immunodeficiencies experience a markedly more severe course of RSV infection [9].

Environmental factors further influence the risk of infection, including exposure to tobacco smoke, which damages the respiratory epithelium and increases susceptibility to respiratory infections [10]. Low socioeconomic status is also relevant, as it correlates with household overcrowding, poor hygienic conditions, and limited access to preventive measures and medical care [11]. Attendance at nurseries and kindergartens additionally increases the risk of infection due to frequent contact with peers and underdeveloped hygienic habits [12].

The clinical manifestations of RSV infection depend on the patient’s age, disease severity, and the presence of comorbidities. In the early stage, nonspecific symptoms such as nasal congestion and cough predominate, often accompanied by fever and lethargy [13,14,15]. As the disease progresses, wheezing develops, indicating airway obstruction, along with tachypnea and increased work of breathing, visible as chest wall retractions and intercostal indrawing [14]. Infants with RSV infection frequently experience feeding difficulties, as dyspnea and nasal congestion interfere with sucking and swallowing, leading to rapid dehydration and weight loss [16]. In more severe cases, cyanosis of the lips and nails is observed as a sign of hypoxemia, requiring immediate medical intervention [17]. In older children, RSV infection is usually milder, although it may still result in significant complications, particularly in patients with pre-existing respiratory conditions.

Diagnosis is based on a thorough physical examination and analysis of clinical symptoms, with particular attention on respiratory function and oxygenation status [18]. Laboratory confirmation is achieved using tests that detect the virus in respiratory specimens, most commonly direct immunofluorescence assays or highly sensitive and specific molecular PCR tests [19]. Imaging studies, such as chest radiography, are performed when complications, especially pneumonia, are suspected [20]. A particularly important component of the diagnostic process is the collection of a detailed medical history, including information on chronic diseases, previous hospitalizations, contact with individuals with infection, and the course of symptoms, including the nature of the cough, breathing pattern, and exercise tolerance. These data form the basis for selecting the optimal therapeutic strategy.

Complications of RSV infection are the main factors determining the length of hospital stay and the complexity of therapeutic management. The most common complications include bronchiolitis and pneumonia, the course of which requires intensive clinical monitoring due to the risk of hypoxemia and progression to respiratory failure [21]. RSV infection carries a high risk of multiple complications, with bronchiolitis being among the most frequently observed, leading to airway obstruction and significant breathing difficulties. The disease may also extend to lung tissue, causing pneumonia manifested by fever, cough, chest pain, and a marked decrease in oxygen saturation [22]. Another common complication is acute otitis media, which particularly affects infants due to the anatomical characteristics of their auditory pathways [23].

In the most severe cases, respiratory failure and shock may develop, posing a life-threatening condition that requires immediate hospitalization and intensive care [24]. In some patients, prolonged bronchial hyperreactivity occurs, increasing the risk of asthma development and recurrent respiratory tract infections. Cases of bronchial fibrosis have also been reported, leading to permanent impairment of respiratory function and a significant reduction in quality of life in later childhood [25].

Preventive measures are a key element in reducing the number of hospitalizations. The most important actions include hand hygiene, surface disinfection, limiting contact with individuals with infection, the use of face masks in the presence of infectious symptoms, and avoidance of environments conducive to viral transmission [26]. Eliminating children’s exposure to tobacco smoke and educating parents on preventive strategies against respiratory infections are of crucial importance. In children at the highest risk, passive immunoprophylaxis with palivizumab is used, which reduces the rate of hospitalization and severe disease [27]. In Poland, a nationwide drug program for RSV prevention is implemented for preterm infants and children with significant comorbidities. In addition, the vaccines Arexvy and Abrysvo have been approved for use in older adults and pregnant women; by inducing maternal antibody production, they provide protection to newborns during the first months of life [28]. The role of nursing staff in the management of RSV infection is indispensable and includes comprehensive monitoring of the child’s vital parameters. Nursing responsibilities involve regular assessment of body temperature, respiratory rate, oxygen saturation, and hydration status, as well as observation of cough, dyspnea, restlessness, and behavioral changes. Owing to their continuous contact with the patient, nurses are often the first to detect signs of clinical deterioration and promptly notify the physician. Emotional support is also fundamental, as a child’s illness generates anxiety and stress in parents, while the child often experiences distress related to hospitalization. The empathetic presence of nursing staff facilitates family adaptation to the illness situation and improves comfort during treatment, thereby contributing to greater therapeutic effectiveness [29]. Both the international literature and Polish literature provide numerous publications describing clinical scenarios associated with RSV infection. Early diagnosis and the implementation of appropriate treatment for RSV infection remain a significant challenge in pediatrics, particularly among the youngest patients. The analysis of factors influencing the course of hospitalization in pediatric patients is therefore crucial for the development of effective therapeutic strategies.

## 2. Materials and Methods

### 2.1. Study Design

A retrospective, analytical observational study was conducted in the Department of Pediatrics, Hospital in Wadowice, Karmelicka 5, 34-100 Wadowice, Poland. Medical records of pediatric patients hospitalized between 29 December 2021 and 14 April 2023 were reviewed. The study was non-interventional in nature and relied exclusively on the analysis of data obtained from patients’ medical histories. During the specified study period, 100 cases of RSV infection were recorded, the medical records of which were included in the study.

### 2.2. Study Material

The study material consisted of medical documentation from 100 pediatric patients with laboratory-confirmed RSV infection. The study included children aged ≤ 5 years with laboratory-confirmed RSV infection who were hospitalized during the study period. RSV infection was confirmed using a rapid antigen detection test performed on nasopharyngeal swab specimens upon admission. Inclusion criteria were: (1) age 0–18 years and (2) laboratory-confirmed RSV infection. Exclusion criteria included congenital heart disease, chronic lung disease (including cystic fibrosis), immunodeficiency, and other severe chronic conditions. Only the first hospitalization of each patient during the study period was included in the analysis.

The following data were extracted from the medical records: patient age; mode of delivery (vaginal delivery vs. cesarean section); gestational age at birth (term birth vs. preterm birth); immunization status (compliant vs. non-compliant with the mandatory vaccination schedule); clinical course of the disease (fever, cough, dyspnea, rhinorrhea); length of hospital stay; need for antibiotic therapy during the course of infection; C-reactive protein (CRP) level; body mass index (BMI); and season of infection (autumn vs. winter) (Table 1).

Patients were assigned to appropriate groups according to the predefined variables:

- Age groups defined based on age ranges established prior to analysis;
- Vaginal delivery vs. cesarean section;
- Term-born (between 37 and 42 weeks of pregnancy) vs. preterm children (before 37 weeks of pregnancy);
- Vaccinated according to the immunization schedule vs. vaccination delays or omissions;
- Infection occurring in autumn vs. winter.

### 2.3. Research Questions

The study assessed associations between selected clinical and demographic variables by addressing the following research questions:

1. Does the mode of delivery (vaginal vs. cesarean section) affect the length of hospitalization due to RSV infection?
2. Does immunization status (compliant vs. non-compliant with the vaccination schedule) influence the length of hospital stay?
3. Does gestational age at birth (term vs. preterm) affect the number of clinical symptoms presented?
4. Do age groups differ in terms of length of hospitalization?
5. Do age groups differ in CRP levels?
6. Does the season of infection (autumn vs. winter) affect the need for antibiotic therapy?
7. Does gestational age at birth (preterm vs. term) influence BMI values in hospitalized patients?
8. Do age groups differ in the number of clinical symptoms (fever, dyspnea, cough, rhinorrhea)?

#### 2.3.1. Data Analysis Methods

Statistical analysis was performed using methods appropriate for categorical and quantitative data. Normality of distribution was checked with the Shapiro–Wilk test in all cases, and the use of subsequent tests results from the normality or otherwise of the distribution. For comparisons of quantitative variables (length of hospitalization, CRP level, BMI) between two groups, the Mann–Whitney U test or Student’s *t*-test was applied, depending on the fulfillment of assumptions regarding normal distribution. For multigroup comparisons (e.g., between age groups), analysis of variance (ANOVA) or its non-parametric equivalent, the Kruskal–Wallis test, was used. Associations between nominal variables (e.g., the need for antibiotic therapy) were analyzed using the chi-square test or Fisher’s exact test, as appropriate. A *p*-value < 0.05 was considered statistically significant. PQStat v.1.8.6 (Poland) was used to perform statistical analyses.

#### 2.3.2. Ethical Considerations

The study was conducted in accordance with the principles of the Declaration of Helsinki. All medical records were anonymized prior to analysis.

## 3. Results

Data from 100 pediatric patients hospitalized with laboratory-confirmed RSV infection were analyzed. The results are presented in relation to the previously formulated research questions. The figures illustrate the observed associations, while a detailed quantitative and qualitative description is provided below.

**Figure 1:**
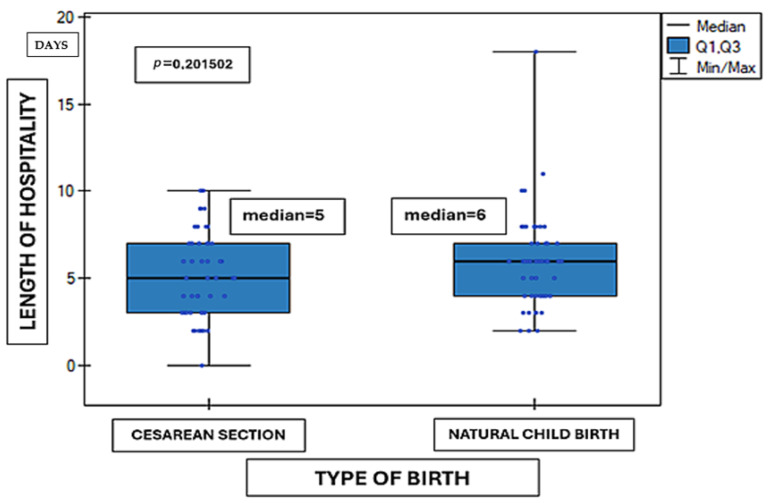
Length of hospital stay according to type of birth (*n* = 97). Data are presented as median (Q1–Q3, minimum–maximum). Statistical analysis: Mann–Whitney U test. Units: days. *p* = 0.2015.

The analysis of the relationship between length of hospital stay and mode of delivery (vaginal delivery versus cesarean section) revealed no statistically significant differences between the studied groups. The mean duration of hospitalization was comparable between children born vaginally and those delivered by cesarean section (Mann–Whitney U test, *p* = 0.2015). Detailed data are presented in Figure 1. In the above analysis, 97 results were included. Lack of research material regarding the mode of delivery concerned three cases.

**Figure 2:**
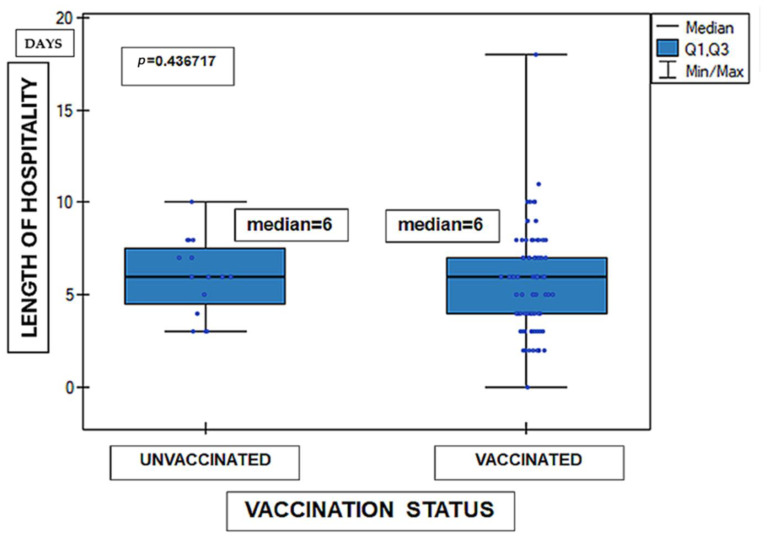
Length of hospital stay according to vaccination status (*n* = 99). Data are presented as median (Q1–Q3, minimum–maximum). Statistical analysis: Mann–Whitney U test. Units: days. *p* = 0.4437.

No statistically significant differences in the length of hospital stay were observed between children vaccinated according to the routine immunization schedule and unvaccinated children (Mann–Whitney U test, *p* = 0.4437). The distribution of hospitalization duration was similar in both groups. The results are presented in Figure 2. In the analysis, 99 results were included. Lack of research material regarding vaccination status concerned one case.

**Figure 3:**
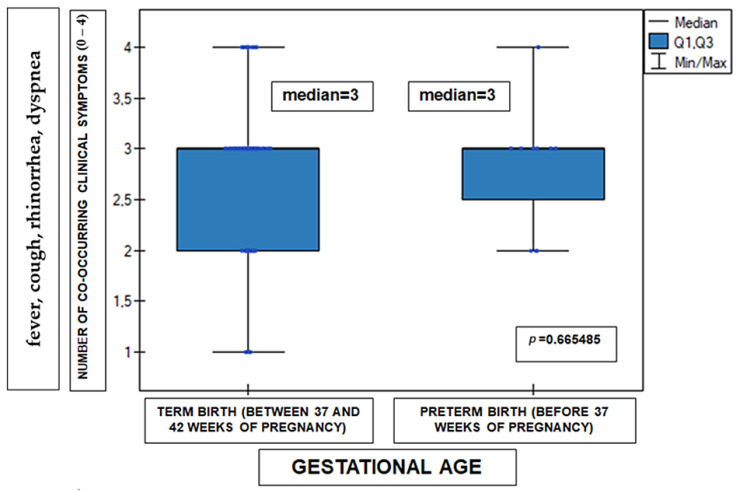
Number of co-occurring clinical symptoms (fever, cough, rhinorrhea, dyspnea; scored 0–4) in infants according to gestational age (*n* = 100). Data are presented as median, interquartile range (Q1–Q3), and minimum–maximum values. Term birth (37–42 weeks of gestation) and preterm birth (<37 weeks of gestation) groups are shown. Median values: 3 for both groups. Statistical comparison was performed using the Mann–Whitney U test; *p* = 0.6655. Units represent the number of co-occurring symptoms.

Figure 3 presents the number of co-occurring clinical symptoms in patients infected with RSV according to gestational age at birth (term-born versus preterm infants). The number of symptoms was calculated as the sum of present clinical symptoms (fever, cough, rhinorrhea, and dyspnea), coded as binary variables (0 = symptom absent, 1 = symptom present). The maximum possible value of the variable was 4. Analysis of the number of co-occurring clinical symptoms, calculated as the sum of reported symptoms (fever, cough, rhinorrhea, and dyspnea), revealed no statistically significant differences between term-born children and preterm infants. The distribution of this variable was comparable in both groups (Mann–Whitney U test, *p* = 0.6655). This relationship is presented in Figure 3. The research material was complete in this respect for all cases and concerned 100 subjects.

**Figure 4:**
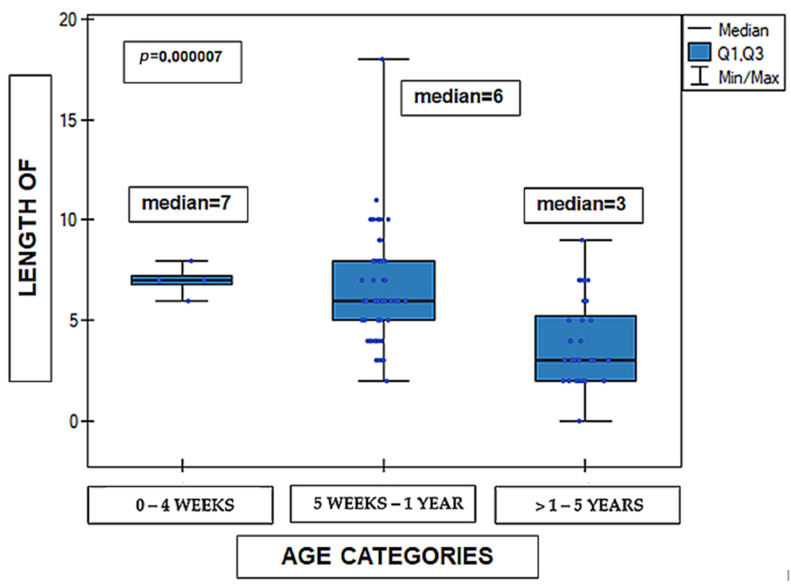
Length of hospital stay (days) according to age category: 0–4 weeks, 5 weeks–1 year, and >1–5 years (*n* = 99). Data are presented as median (line within the box), interquartile range Q1–Q3 (box), and minimum–maximum values (whiskers); individual dots represent single observations. Differences between groups were assessed using the Kruskal–Wallis test. *p* = 0.000007.

Statistically significant differences in the length of hospital stay were observed between the individual age groups. The longest duration of hospitalization was noted in the youngest age groups, whereas shorter hospital stays were observed in older children (Kruskal–Wallis test, *p* < 0.00001). The material concerned 99 cases in terms of age, because one of the cases was excluded from the analysis due to the patient’s age being greater than 5 years (12 years). A detailed distribution of the data is presented in Figure 4.

**Figure 5:**
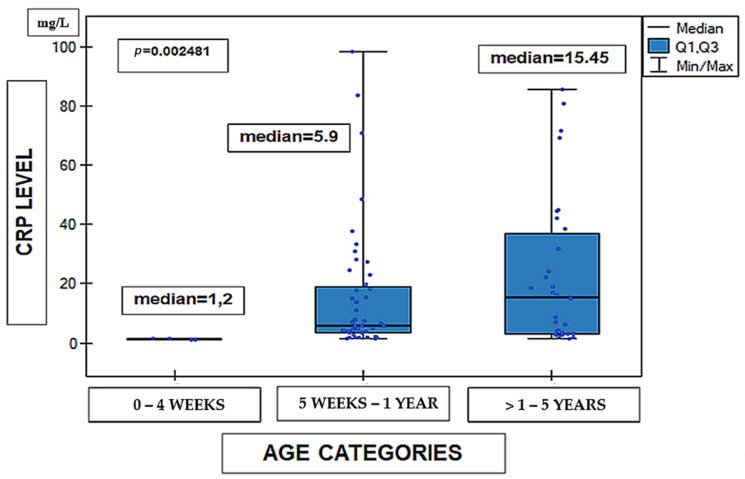
C-reactive protein (CRP) levels (mg/L) according to age category (*n* = 99). Data are presented as median (line within the box), interquartile range Q1–Q3 (box), and minimum–maximum values (whiskers); individual dots represent single observations. Differences between groups were assessed using the Kruskal–Wallis test. *p* = 0.0025.

Analysis of C-reactive protein (CRP) levels revealed statistically significant differences between age groups. CRP concentrations varied according to patient age (Kruskal–Wallis test, *p* = 0.0025), as illustrated in Figure 5. The material concerned 99 cases in terms of age, because one of the cases was excluded from the analysis due to the patient’s age being greater than 5 years (12 years).

**Figure 6:**
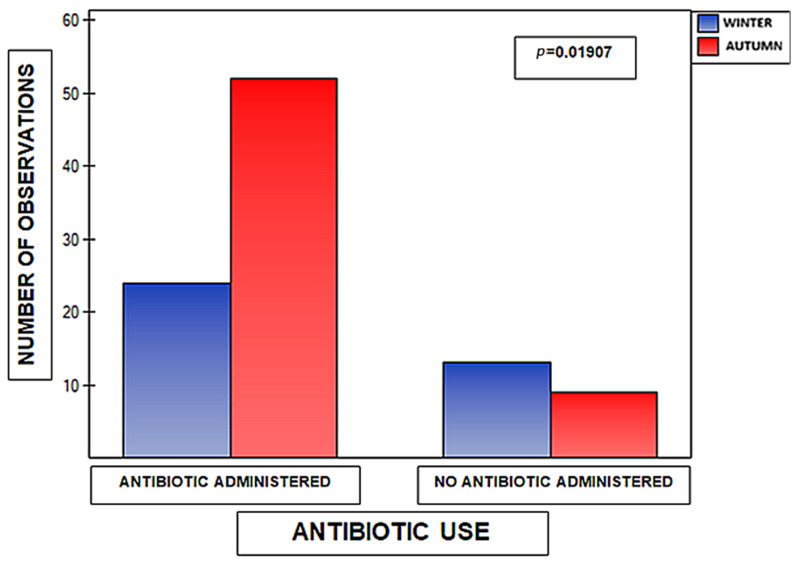
Seasonal distribution of antibiotic use (*n* = 100). The number of observations with antibiotics administered and not administered is shown for winter (blue) and autumn (red). Data are presented as absolute counts. Differences in antibiotic use between seasons were assessed using the chi-square test. *p* = 0.0191.

A statistically significant association was demonstrated between the season of infection and the need for antibiotic therapy. The proportion of patients in whom antibiotic treatment was initiated differed between the autumn and winter seasons (chi-square test, *p* = 0.0191). The results of this analysis are presented in Figure 6. The research material was complete in this respect for all cases and concerned 100 subjects.

Analysis of body mass index (BMI) revealed statistically significant differences between preterm-born children and those born at term (*p* = 0.028096).

**Figure 7:**
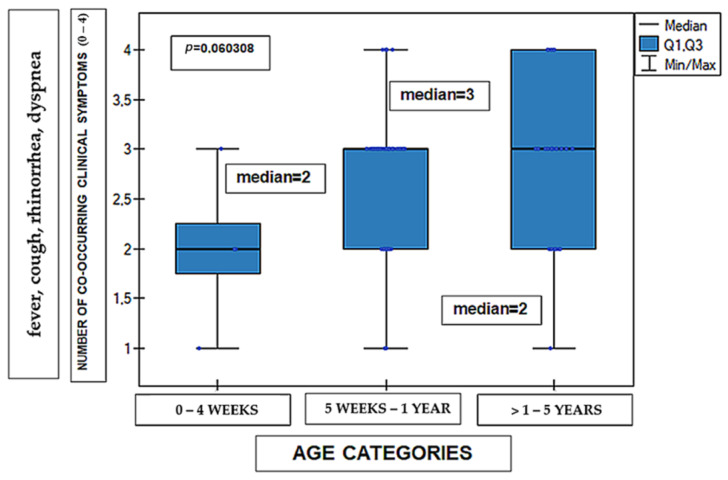
The dependence of the number of clinical symptoms on age group (n = 99). Data are presented as median (line within the box), interquartile range Q1–Q3 (box), and minimum–maximum values (whiskers); individual dots represent single observations. Differences between groups were assessed using the Kruskal–Wallis test. *p* =0.0603.

Analysis of the number of co-occurring clinical symptoms, calculated as the sum of present symptoms (fever, cough, rhinorrhea, and dyspnea), revealed no statistically significant differences between the analyzed age groups. The distribution of this variable was comparable across all groups (Kruskal–Wallis test, *p* = 0.0603). The distribution of symptom counts is presented in Figure 7. The material concerned 99 cases in terms of age, because one of the cases was excluded from the analysis due to the patient’s age being greater than 5 years (12 years).

The figure presents the number of co-occurring clinical symptoms in patients infected with RSV according to age group. The number of symptoms was calculated as the sum of present clinical symptoms (fever, cough, rhinorrhea, and dyspnea), coded as binary variables (0 = symptom absent, 1 = symptom present). The maximum possible value of the variable was 4.

## 4. Discussion

This study provides insights into the factors influencing the course of hospitalization of children infected with RSV in a pediatric ward setting, highlighting the significance of patient age, infection seasonality, and selected clinical parameters, while demonstrating no effect of certain commonly analyzed perinatal factors. A particularly important and original aspect of this work is the comprehensive, simultaneous analysis of multiple clinical and demographic variables within a homogeneous pediatric population hospitalized at a single center, allowing for coherent and practically applicable conclusions that reflect real-world clinical conditions. Importantly, this approach allows identification of factors influencing the in-hospital course of RSV infection rather than the risk of infection itself.

One of the key findings was a significant association between the child’s age and both the length of hospital stay and the intensity of the inflammatory response, as measured by CRP levels. The youngest age groups were characterized by longer hospitalization and higher inflammatory marker values, confirming the particular vulnerability of infants to a more severe course of RSV infection. These findings have direct clinical implications, emphasizing the need for more intensive monitoring of the youngest patients. From a practical perspective, these results support age-oriented triage and monitoring strategies in pediatric wards, with particular attention on infants requiring prolonged hospitalization and closer observation.

An interesting and clinically relevant observation was the lack of influence of the mode of delivery and adherence to the routine immunization schedule on the length of hospitalization. This result may indicate that, in the context of RSV infection, these factors do not play a key role in the course of acute hospitalization, in contrast to patient age and the infectious season. This observation distinguishes the present study from some reports suggesting a potential impact of perinatal factors on the risk of severe respiratory tract infections and provides a basis for further analyses in larger populations.

The findings related to infection seasonality and the associated frequency of antibiotic use are particularly noteworthy in the context of current challenges related to the rational use of antibiotics. The demonstrated significant difference between the autumn and winter seasons may suggest the influence of epidemiological, organizational, or diagnostic factors on therapeutic decision-making, warranting further investigation and potentially serving as an impetus for the development of season-specific clinical management algorithms. These findings suggest that external epidemiological context, rather than objective markers of disease severity alone, may influence antibiotic prescribing practices during RSV seasons.

The obtained results raise new research questions, including those involving the role of seasonality in therapeutic decision-making, the influence of patient age on hospitalization strategies, and the potential development of predictive tools to support patient selection for hospitalization during the course of RSV infection.

Referring to the selected literature, it is worth mentioning the study by Cegielska et al., who presented research conducted in 2018 involving a total of 71 patients with RSV infection [30]. The aim of their study was to analyze the clinical course of RSV infection in children up to 24 months of age treated during the 2016–2017 season. Their findings demonstrated that the average length of hospitalization was 8 days. In contrast, in the present study involving 100 pediatric patients with RSV infection, the mean duration of hospital stay was 6 days. The authors also observed an increase in the inflammatory marker (CRP) among patients with infection. Similarly, in the present study, CRP levels ranged between 1 and 20 mg/L in 60% of patients; in 18 patients, CRP values were <1 mg/L, whereas in 5% of cases, CRP levels were markedly elevated, exceeding 80 mg/L.

Another relevant study is that by Nitsch-Osuch et al., who described RSV infections in relation to seasonal patterns influenced by geographical conditions. Based on their observations, in the Northern Hemisphere, the RSV season typically extends from autumn to spring, with a peak incidence in January and February. However, due to the COVID-19 pandemic and the preventive measures implemented at that time, this pattern was disrupted, and recent years have shown an increased incidence of RSV infections outside the typical seasonal period for a given region. In the present study, 61% of RSV-related hospitalizations occurred in autumn, while 37% were recorded in winter, and only 2% were recorded in spring and summer combined. In contrast, the authors noted that virtually all children up to 2 years of age experience RSV infection at some point. They also demonstrated a causal association between RSV infection and the development of lower respiratory tract infections in children younger than 1 year. The need for hospital treatment was particularly evident in children under 5 years of age due to significant symptom severity and its impact on overall health status [31]. According to the present data analysis, the largest proportion of hospitalized patients with RSV infection was infants aged between 5 weeks and 1 year. In another review article by Kaler et al., the issue of prematurity as a risk factor for increased susceptibility to RSV infection was addressed. According to the authors, this is primarily due to the immaturity of the immune system resulting from insufficient passive immunity conferred by maternal antibodies, which play a critical role in protecting against infections during the first months of life. Given the reduced immune competence observed in preterm infants, this group is at a significantly higher risk of severe disease manifestations compared with older children and adults. In the present study, only 15% of children were born preterm, while 85% were born at term. No statistically significant differences were observed between term-born and preterm children with regard to the length of hospitalization due to RSV infection. It is also noteworthy that the authors of the aforementioned review reported that most patients initially presented with mild-to-moderate rhinorrhea, low-grade fever, and a productive cough developing several days after infection.

Furthermore, the study highlighted that infants and children with RSV infection may present with a wide spectrum of clinical manifestations, ranging from mild upper respiratory tract symptoms to life-threatening lower respiratory tract involvement requiring specialized treatment, including mechanical ventilation. In the present study, fever was observed in 52% of patients, while 48% did not present with elevated body temperature. Rhinorrhea was reported in 75% of patients, whereas 25% did not exhibit this symptom. Cough was present in 97% of cases, while only 3% of patients did not report coughing. An additional noteworthy issue addressed in the reviewed article concerns the use of antibiotic therapy in RSV infection. According to the authors, antibiotics are not considered first-line treatment for RSV infection, and the decision to initiate antibiotic therapy depends on the patient’s clinical presentation, particularly when bacterial superinfection is suspected. Laboratory findings guiding antibiotic use typically include elevated serum CRP levels; therefore, antibiotic therapy is generally limited to children who exhibit unexpected clinical deterioration [32]. In the present study, slightly more than three-quarters of patients received antibiotic therapy during hospitalization.

Previous studies have consistently demonstrated that immunocompromised children represent a population at particularly high risk for severe RSV infection, frequent hospitalization, and adverse clinical consequences, including disruptions to the treatment of underlying diseases. In the study group described by the authors from St. Jude Children’s Research Hospital, RSV infection was associated not only with a substantial hospitalization rate but also with clinically significant delays or modifications in oncological therapy in nearly one third of patients, underscoring the broader systemic impact of RSV in immunodeficient populations. In contrast, the present study focused on immunocompetent pediatric patients, the majority of whom were vaccinated according to the routine immunization schedule and without documented immune deficiencies. In this cohort, disease course and length of hospitalization were primarily determined by patient age and infection seasonality rather than by immunological vulnerability. The absence of severe systemic consequences, such as treatment delays or increased mortality, differentiates immunocompetent children from immunocompromised patients and highlights the heterogeneity of RSV infection outcomes. These findings emphasize that while RSV poses a significant burden across pediatric populations, the mechanisms driving disease severity and clinical decision-making differ substantially depending on immune status, reinforcing the need for stratified risk assessment and tailored management strategies [33].

Large population-based studies have demonstrated that young age, particularly infancy, is a major determinant of disease severity in RSV-associated hospitalizations. In one study group comprising 219,997 children under 24 months of age, infants younger than 6 months exhibited the highest hospitalization rates due to RSV and significantly increased risks of intensive care unit admission, respiratory support, and complex hospital courses compared with older infants and children infected with other respiratory viruses. These findings are consistent with the results of the present study, which also identified patient age as a key factor influencing the clinical course of RSV infection. In our study group, the youngest age groups were characterized by significantly longer lengths of hospital stay and higher levels of systemic inflammatory response, as reflected by elevated C-reactive protein concentrations. While the referenced population-based analysis focused on severe clinical endpoints such as ICU admission and respiratory support in early infancy, our findings extend this evidence by demonstrating that even within a broader pediatric population, younger age is associated with increased inflammatory burden and prolonged hospitalization. Notably, unlike age and infection seasonality, perinatal factors such as mode of delivery and adherence to the routine immunization schedule did not significantly affect hospitalization duration in our cohort, underscoring the predominant role of age-related physiological and immunological vulnerability in shaping the acute course of RSV infection [34].

Taken together, our findings indicate that in immunocompetent pediatric patients hospitalized with RSV infection, age-related physiological vulnerability outweighs perinatal history in determining the course of hospitalization. This observation may help refine clinical decision-making by prioritizing current clinical status and age over historical perinatal factors.

## 5. Conclusions

The mode of delivery did not affect the length of hospitalization in children with acute RSV infection, indicating that delivery-related perinatal factors should not be considered determinants of hospital stay duration. Likewise, adherence to the routine vaccination schedule was not associated with hospitalization length, suggesting that the in-hospital course of RSV infection should be guided primarily by the child’s current clinical condition and age rather than vaccination history.

No significant differences in symptom severity were observed between children born at term and those born preterm, highlighting the importance of individualized clinical assessment, as prematurity alone does not predict a more symptomatic acute course. In contrast, age was a significant factor influencing hospitalization duration, with younger children more likely to require prolonged inpatient care and closer monitoring.

Significant age-related differences were also noted in inflammatory response, as reflected by CRP concentrations, indicating greater susceptibility of younger patients to systemic inflammation. Seasonal variations in antibiotic use suggest that external epidemiological and organizational factors influence treatment decisions, underscoring the need for strengthened antimicrobial stewardship, particularly during peak RSV seasons.

Differences in BMI between term and preterm children likely reflect divergent growth and nutritional trajectories, supporting the routine assessment of nutritional status during hospitalization, especially in preterm-born patients. Finally, the absence of age-related differences in the number of observed symptoms suggests that symptom count alone is not a reliable indicator of disease severity and should not be used as the sole basis for clinical decision-making.

### Study Limitations

This study was conducted at a single pediatric center, which may limit the generalizability of the findings to other clinical settings or healthcare systems. Therefore, the results should be interpreted with caution, and confirmation in multicenter studies would be valuable.

BMI in preterm infants is highly age-dependent and may change substantially over short periods of time. The lack of age-stratified BMI analysis represents a limitation of the present study and should be considered when interpreting the results. Another limitation of the study is the lack of data on maternal vaccination status, which should be considered in future research, as including this variable may provide a more comprehensive assessment of immunity-related factors in newborns. The study did not collect detailed information on passive smoking exposure (e.g., any smoking in the household, indoor smoking, or the number of cigarettes smoked per day by parents), which should be considered in future research as a potential factor influencing hospitalization outcomes.

## Figures and Tables

**Table 1:** Baseline characteristics of the study population.

Variable	Value
(Number of patients n = 100)	
Age	
0–4 weeks, *n* (%)	4 (4.0%)
5 weeks–1 year, *n* (%)	62 (62.0%)
>1–5 years, *n* (%)	33 (33.0%)
Mode of delivery	
Natural child birth, *n* (%)	49 (49.0%)
Cesarean section, *n* (%)	48 (48.0%)
Immunization status	
Vaccinated	84 (84.0%)
Unvaccinated	15 (15.0%)
Clinical symptoms	
Fever, *n* (%)	52 (52.0%)
Cough, *n* (%)	97 (97.0%)
Dyspnea, *n* (%)	54 (54.0%)
Rhinorrhea, *n* (%)	75 (75.0%)
Length of hospital stay (days)	median = 6 (0–18)
Antibiotic therapy	
Yes, *n* (%)	76 (76.0%)
No, n (%)	24 (24.0%)
Season of RSV infection	
Autumn, *n* (%)	62 (62.0%)
Winter, *n* (%)	32 (32.0%)

Percentages may not sum to 100% due to missing or incomplete data in selected variables. Percentages were calculated based on available data for each variable.

## Data Availability

The data presented in this study are available on request from the Dominik Gałuszka due to collaboration agreements with the hospital unit where the research was conducted.
